# Does poor oral health status increase the risk of falls?: The JAGES Project Longitudinal Study

**DOI:** 10.1371/journal.pone.0192251

**Published:** 2018-02-01

**Authors:** Yuki Mochida, Tatsuo Yamamoto, Shinya Fuchida, Jun Aida, Katsunori Kondo

**Affiliations:** 1 Division of Dental Sociology, Department of Oral Science, Graduate School of Dentistry, Kanagawa Dental University, Yokosuka, Japan; 2 Department of International and Community Oral Health, Tohoku University Graduate School of Dentistry, Sendai, Japan; 3 Center for Preventive Medical Sciences, Chiba University, Chiba, Japan; 4 Center for Gerontology and Social Science, National Center for Geriatrics and Gerontology, Obu, Japan; 5 Center for Well–being and Society, Nihon Fukushi University, Nagoya, Japan; Virginia Commonwealth University, UNITED STATES

## Abstract

We sought to examine if self-reported oral health conditions regarding difficulty eating tough foods, dry mouth, choking, number of teeth and denture use are associated with incident falls. Our study was based on panel data from the Japan Gerontological Evaluation Study conducted in 2010 and 2013 using self-administered questionnaires. Data from 19,995 male and 20,858 female community-dwelling older people aged ≥65 years without a history of falls within the previous year in 2010 were analyzed. Multilevel logistic regression models were used to determine the association between poor oral health in 2010 and multiple incident falls in 2013 after adjusting for possible confounders and considering differences in municipalities. The percentage of males and females who reported falls in 2013 were 2.4% and 2.1%, respectively. After adjusting for age, educational attainment, equivalized income, depression, self-rated health, instrumental activities of daily living, body mass index, present illness related to falls, social participation, walking in min/day, alcohol drinking status, and municipality population density, dry mouth in males (odds ratio [OR] = 1.41; 95% confidence interval [CI]: 1.12–1.77) and choking in females (OR = 1.64; 95% CI: 1.27–2.11) were significantly associated with incident falls. Difficulty eating tough foods in both sexes and choking in males were marginally associated with incident falls (p<0.1). Females having 10–19 teeth without dentures (OR = 1.63; 95% CI: 1.14–2.31), ≤9 teeth with dentures (OR = 1.36; 95% CI: 1.03–1.80), and ≤9 without dentures (OR = 1.46; 95% CI: 1.02–2.08) were significantly associated with incident falls compared with those having ≥20 teeth, respectively. These findings suggest that poor oral function, having fewer teeth, and not using dentures are predictors of incident falls. Further studies are needed to determine whether improving oral health can reduce the risk of falls.

## Introduction

As the world’s population continues to age, falls are becoming an increasingly major public health problem. In England, it has been reported that approximately 28–35% of older people aged ≥65 years experienced a fall in the previous year [1,2]. Injuries from falls can range from light bruises to hip fractures, and can even result in death [3]. At least 10% of fallers in the U.S. experienced serious injuries [4]. In addition, falls and fractures account for 12.2% of all causes leading to the need for long-term care among older people in Japan [5].

It is therefore important to identify risk factors for falls. Although little can be done about biological risk factors such as age, sex, and chronic illness, behavioral risk factors such as lack of exercise and medication use can be modified by interventions and changes in individual behavior [6]. One recent study revealed that having a low number of teeth and not using dentures were associated with a higher frequency of falls [7]. A longitudinal study involving 4,425 community-dwelling older people showed that those having ≤19 teeth without dentures had a significantly higher risk for incident falls than those having ≥20 teeth [7].

However, the association between oral function and falls remains controversial. One cross-sectional study involving 87 older people showed an association between a decrease in occlusal function and postural instability [8]. Another longitudinal study involving 348 older people showed that occlusal disharmony is a risk factor for a decrease in balance function [9]. A cross-sectional study involving 34 frail older people showed that lower occlusal force was associated with a higher risk of falls, as assessed using a 21-item fall risk index [10]. On the other hand, a longitudinal study found no significant associations between self-reported chewing ability and incident falls [7].

Swallowing is another oral function closely linked to chewing ability because it follows mastication and bolus formation. Saliva production also contributes to mastication, bolus formation, and swallowing [11]. Questions regarding three oral functions, difficulty eating tough foods, dry mouth and choking are included on the basic screening checklist for frail older people in the Japanese long-term care insurance system [12]. A cross-sectional study reported that these questions were significantly associated with falls [13]; however, their temporal relationship remains unknown. If a decline in oral function is found to precede falls, it could become an accurate predictor of falls and be useful for collaborating oral health and fall prevention in older people from the perspective of health policy.

Therefore, the present study investigated the association between oral function and incident falls using longitudinal data from community-dwelling older people. In addition, it has been suggested that falls show regional differences [14], so the association between number of teeth and/or denture use and incident falls was also investigated to verify the reproducibility of the results of a previous study [7] using a large sample and multilevel logistic regression models.

## Materials and methods

### Study population

Data from a longitudinal study, collected as part of the Japan Gerontological Evaluation Study (JAGES) project, an ongoing Japanese prospective cohort study [15,16], were used for the present study. The JAGES aims to investigate factors associated with the loss of healthy years, such as functional decline, cognitive impairment, and death among non-institutionalized older people. The JAGES sample was restricted to those who did not already have physical or cognitive disability at baseline, which was defined as not receiving long-term public care insurance benefits and having self-reported dependence in walking, toileting, and bathing.

**Fig 1:**
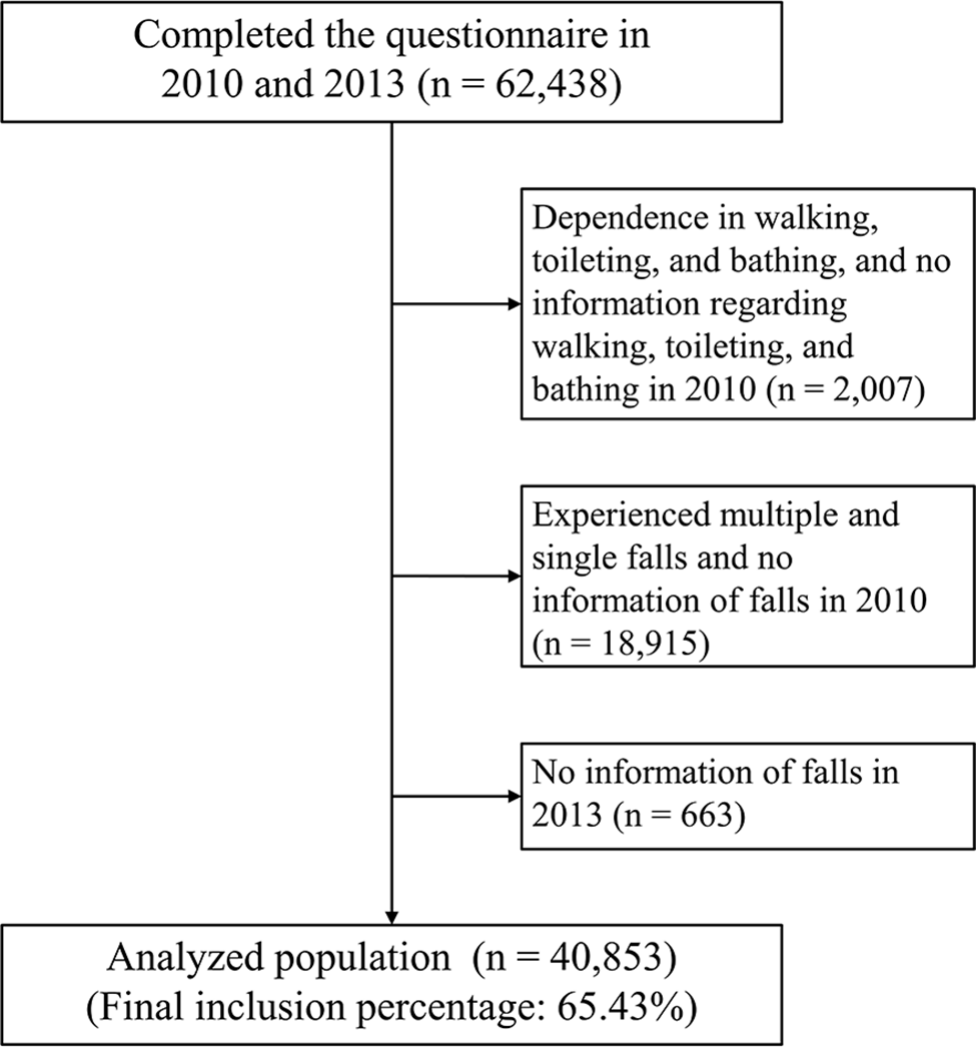
Flow chart of the participant selection process.

Our analyses used the panel data from two surveys. The baseline survey was conducted between August 2010 and January 2012 among 141,452 people aged ≥65 years. Self-administered questionnaires were mailed to the entire population of 10 municipalities, and randomly to selected residents in 14 municipalities based on the official residential registers obtained from the respective municipal governments. A total of 92,272 people responded to the questionnaires (response rate: 65.2%). A follow-up survey using self-administered questionnaires was conducted between October 2013 and December 2013 on the same respondents in the same municipalities. Collectively, 62,438 individuals completed both the 2010 and 2013 questionnaires. Data from 40,853 respondents (19,995 males and 20,858 females) were used for the analyses after excluding those from 2,007 who already had self-reported dependence in walking, toileting, and bathing at baseline, 16,240 who had experienced single or multiple falls at baseline, 2,675 who provided no information on falls at baseline, and 663 who provided no information on falls at follow-up (Fig 1. Flow chart of the participant selection process). The JAGES protocol was reviewed and approved by the Nihon Fukushi University Ethics Committee (No. 10–05), and the Ethical Committee of Kanagawa Dental University (No. 466) approved the analysis of data in the present study.

### Outcome variables

The incidence of falls was determined by asking, “How many times have you fallen within the past year?”, with possible answers of “multiple times”, “once”, or “none”. Multiple falls was utilized as an outcome after combining the last two categories because previous studies have shown that single fallers are more similar to nonfallers than to recurrent fallers on a range of medical, physical, and psychological risk factors [17–19].

### Oral health variables

Oral function, including difficulty eating tough foods, dry mouth, and choking, and dental status were assessed using self-administered questionnaires. Difficulty eating tough foods, dry mouth, and choking were determined by asking, “Do you have any difficulties eating tough foods now compared with 6 months ago?”, “Do you often have dry mouth?”, and “Have you recently choked on your tea or soup?”, respectively, with possible answers dichotomized into yes and no, as utilized in a basic checklist for nursing care prevention in the Japanese long-term care insurance system [12]. Dental status was categorized as follows: having ≥20 teeth, having 10–19 teeth with dentures, having 10–19 teeth without dentures, having ≤9 teeth with dentures, and having ≤9 teeth without dentures.

### Covariates

Factors associated with falls, including age [7,20,21], educational attainment [7,22], equivalized income [22,23], depression [7,21], self-rated health [7,24], instrumental activities of daily living (IADL) [13], body mass index (BMI) [24], present illness related to falls [20,21], social participation [22], walking in min/day [22], alcohol drinking status [25] and population density [22], were used as covariates. Age was categorized as follows: 65–69, 70–74, 75–79, 80–84 or ≥85 years. Educational attainment was categorized as follows: ≤9, 10–12, or ≥13 years. Equivalized income was calculated by dividing household income by the square root of the number of household members, and was categorized as follows: ≤1,999,999 JPY (1 USD = 100 JPY), 2,000,000–3,999,999 JPY, or ≥4,000,000 JPY. Depression was assessed using the Japanese short version of the Geriatric Depression Scale-15 [26], and was grouped into three categories: 0–4 (no), 5–9 (mild), or 10–15 (moderate to severe). Self-rated health was determined by asking, “How is your health at present?”, with answers categorized as follows: “excellent”, “good”, “fair”, or “poor”. IADL was assessed using the Tokyo Metropolitan Institute of Gerontology Index of Competence (TMIG-IC) questionnaire [27], and categorized as follows: independence (13 points) or dependence (≤12 points). BMI was categorized into three groups: <18.5, 18.5–24.9, or ≥25.0. Self-reported current medical treatment for stroke, osteoporosis, joint disease/neuralgia, injury/fracture, impaired vision and/or impaired hearing was used as a variable for present illness related to falls and categorized into two groups: yes or no. Social participation was determined by asking, “Do you belong to the following organization or group?” in relation to the following types of community organizations: neighborhood/senior association, citizen/firefighting club, religious group, political group/organization, industrial or trade association, volunteer group, citizen/consumer group, hobby group, and sports club/group. Answers were categorized into two groups: participation (yes) or nonparticipation (no). Walking in min/day was categorized as follows: ≥90 min, 60–89 min, 30–59 min, or <30 min. Alcohol drinking status was categorized as follows: current, former, or never drinker. Municipality population density was categorized as follows: metropolitan (density over 4,000 people per km^2^), urban (density between 1,500 and 4,000 people per km^2^), semi-urban (density between 1,000 and 1,499 people per km^2^), and rural (density below 1,000 people per km^2^).

### Statistical analysis

Categorical variables that included missing values were recorded by reassigning missing values to separate “data missing” categories to maximize the number of participants included in the statistical analysis and thereby maximize statistical power. In the follow-up survey, incident falls was defined as a history of multiple falls. First, univariate associations between incident falls and oral health variables and covariates in males and females were examined. Then, two-level (first level: individuals; second level: municipality) logistic regression models with random intercepts and fixed slopes were used for males and females separately to calculate multilevel odds ratios (ORs) and 95% confidence intervals (CIs) for incident falls at follow-up. In the first model, univariate ORs and 95% CIs were calculated for each oral health variable. In the second model, multilevel ORs and 95% CIs were calculated for each oral health variable after adjusting for age. In the third model, multilevel ORs and 95% CIs were calculated for each oral health variable after adjusting for all covariates, i.e., age, educational attainment, equivalized income, depression, self-rated health, IADL, BMI, present illness related to falls, social participation, walking in min/day and alcohol drinking status, as individual-level variables, and population density as a municipality-level variable. In the fourth model, difficulty eating tough foods, dry mouth, and choking were simultaneously added after adjusting for all covariates. In the fifth model, all oral health variables were simultaneously added after adjusting for all covariates and checking the multicollinearity among the oral health variables. All statistical analyses were performed using MLwiN 2.36 (Centre for Multilevel Modelling, University of Bristol, Bristol, UK) and IBM SPSS Statistics (version 23.0; IBM Co., New York, NY, USA).

## Results

The number (%) of males and females who reported having multiple falls in the follow-up survey were 475 (2.4%) and 430 (2.1%), respectively. Table 1 shows the rates of males and female fallers at follow-up according to oral health variables and covariates. In both sexes, participants with difficulty eating tough foods, dry mouth, choking, poor dental status, older age, low educational attainment, low equivalized income, depression, poor self-rated health, low IADL, low BMI, present illness related to falls, social nonparticipation, <30 min walking/day, status as a former drinker and living in rural areas were more likely to report the occurrence of incident falls.

**Table 1:** Univariate associations of oral health variable and covariates with incident falls in males and females.

		Males			Females		
		Total	Fallers		Total	Fallers	
		n	n	%	n	n	%
**Oral health variables**							
Difficulty eating tough foods	No	14749	295	2.00	15786	270	1.71
	Yes	4261	155	3.64	4179	135	3.23
	Data missing	985	25	2.54	893	25	2.80
Dry mouth	No	15793	322	2.04	16480	296	1.80
	Yes	3061	124	4.05	3195	105	3.29
	Data missing	1141	29	2.54	1183	29	2.45
Choking	No	16668	360	2.16	17539	310	1.77
	Yes	2302	93	4.04	2377	90	3.79
	Data missing	1025	22	2.15	942	30	3.18
Dental status	≥20 teeth	7965	130	1.63	7973	105	1.32
	10–19 teeth with dentures	3280	71	2.16	3101	56	1.81
	10–19 teeth without dentures	1707	41	2.40	1891	48	2.54
	≤9 teeth with dentures	4379	129	2.95	4601	134	2.91
	≤9 teeth without dentures	1712	64	3.74	1659	52	3.13
	Data missing	952	40	4.20	1633	35	2.14
**Covariates**							
Age (years)	65–69	7381	100	1.35	7523	78	1.04
	70–74	6224	122	1.96	6571	134	2.04
	75–79	4074	126	3.09	4278	121	2.83
	80–84	1822	85	4.67	1874	70	3.74
	≥85	494	42	8.50	612	27	4.41
Educational attainment (years)	≤9	7694	237	3.08	9603	218	2.27
	10–12	6857	137	2.00	7720	140	1.81
	≥13	4867	75	1.54	2798	41	1.47
	Data missing	577	26	4.51	737	31	4.21
Equivalized income (10,000 yen)	Low (≤199)	7738	215	2.78	8227	168	2.04
	Middle (200–399)	7937	138	1.74	6668	117	1.75
	High (≥400)	2219	45	2.03	1923	29	1.51
	Data missing	2101	77	3.66	4040	116	2.87
Depression	No	13791	262	1.90	13397	212	1.58
	Mild	2997	110	3.67	2945	84	2.85
	Moderate to severe	799	48	6.01	719	33	4.59
	Data missing	2408	55	2.28	3797	101	2.66
Self-rated health	Excellent	3081	40	1.30	2887	32	1.11
	Good	14137	296	2.09	15269	286	1.87
	Fair	2345	114	4.86	2228	92	4.13
	Poor	295	19	6.44	224	18	8.04
	Data missing	137	6	4.38	250	2	0.80
IADL	Independence (13)	7012	134	1.91	10672	152	1.42
	Dependence (≤12)	11209	289	2.58	8157	210	2.57
	Data missing	1774	52	2.93	2029	68	3.35
Body mass index	<18.5	826	28	3.39	1609	35	2.18
	18.5–24.9	13835	319	2.31	14222	248	1.74
	≥25	4640	102	2.20	4026	110	2.73
	Data missing	694	26	3.75	1001	37	3.70
Present illness related to falls*	No	10612	231	2.18	9167	155	1.69
	Yes	3979	150	3.77	6322	204	3.23
	Data missing	5404	94	1.74	5369	71	1.32
Social participation	No	3280	100	3.05	3191	94	2.95
	Yes	14476	293	2.02	14703	258	1.75
	Data missing	2239	82	3.66	2964	78	2.63
Walking in min/day	≥90	3760	73	1.94	3433	58	1.69
	60–89	3634	74	2.04	3199	52	1.63
	30–59	6999	156	2.23	7415	149	2.01
	<30	5319	164	3.08	5976	144	2.41
	Data missing	283	8	2.83	835	27	3.23
Alcohol drinking status	Never drinker	6471	169	2.61	16205	341	2.10
	Former drinker	985	27	2.74	179	9	5.03
	Current drinker	11582	255	2.20	3447	54	1.57
	Data missing	957	24	2.51	1027	26	2.53
Population density (person per square kilometers)	Metropolitan (≥4,000)	5340	109	2.04	5536	86	1.55
	Urban (1,500–3,999)	5079	104	2.05	4913	79	1.61
	Semiurban (1,000–1,499)	4505	117	2.60	4673	97	2.08
	Rural (≤999)	5071	145	2.86	5736	168	2.93

*Stroke, osteoporosis, joint disease/neuralgia, injury/fracture, impaired vision and/or impaired hearing.

Table 2 shows the ORs (95% CIs) for male fallers according to oral health variables in the five multilevel logistic regression models. In Models 1 and 2, all oral health variables were significantly associated with incident falls. In Model 3, the associations between falls and difficulty eating tough foods, dry mouth, and choking remained significant; however, no significant associations were found between dental status and incident falls. In Models 4 and 5, three kinds of difficulties in oral function were associated with incident falls, although the statistical significance was marginal for difficulty eating tough foods in Model 5 and choking in Models 4 and 5 (p<0.10). In Model 5, dry mouth had a significantly high OR (1.41; 95% CI: 1.12–1.77); however, no significant association was observed between dental status and incident falls.

**Table 2:** Odds ratios and their 95% confidence intervals of oral health variables in the adjusted multilevel logistic regression models in males.

		Model 1				Model 2				Model 3				Model 4				Model 5			
		OR	95% CI		p	OR	95% CI		p	OR	95% CI		p	OR	95% CI		p	OR	95% CI		p
			Low	High			Low	High			Low	High			Low	High			Low	High	
Difficulty eating tough foods (reference: No)	Yes	1.86	1.52	2.26	<0.001	1.70	1.39	2.07	<0.001	1.33	1.08	1.64	0.007	1.27	1.03	1.56	0.028	1.23	0.99	1.52	0.060
	Data missing	1.30	0.85	1.97	0.222	1.18	0.78	1.79	0.441	1.02	0.53	1.98	0.945	2.12	0.86	5.21	0.103	1.98	0.80	4.89	0.139
Dry mouth (reference: No)	Yes	2.03	1.64	2.51	<0.001	1.90	1.53	2.35	<0.001	1.49	1.19	1.86	<0.001	1.41	1.12	1.77	0.003	1.41	1.12	1.77	0.003
	Data missing	1.27	0.86	1.87	0.233	1.15	0.78	1.69	0.491	0.89	0.51	1.55	0.684	1.05	0.52	2.10	0.898	1.04	0.52	2.09	0.909
Choking (reference: No)	Yes	1.91	1.51	2.41	<0.001	1.67	1.32	2.11	<0.001	1.37	1.07	1.74	0.012	1.24	0.97	1.59	0.092	1.24	0.97	1.59	0.089
	Data missing	1.01	0.65	1.56	0.982	0.92	0.59	1.43	0.718	0.62	0.32	1.20	0.157	0.37	0.13	1.04	0.058	0.38	0.13	1.06	0.065
Dental status (reference: ≥20 teeth)	10–19 teeth with dentures	1.33	0.99	1.79	0.056	1.26	0.94	1.69	0.219	1.18	0.88	1.59	0.265					1.12	0.83	1.51	0.465
	10–19 teeth without dentures	1.47	1.03	2.10	0.035	1.37	0.96	1.96	0.087	1.16	0.81	1.67	0.410					1.13	0.79	1.63	0.504
	≤9 teeth with dentures	1.81	1.41	2.32	<0.001	1.39	1.08	1.80	0.011	1.18	0.91	1.53	0.203					1.12	0.86	1.46	0.401
	≤9 teeth without dentures	2.30	1.69	3.12	<0.001	1.75	1.28	2.38	<0.001	1.36	0.99	1.87	0.059					1.29	0.93	1.78	0.125
	Data missing	2.71	1.88	3.91	<0.001	2.03	1.40	2.93	<0.001	1.52	1.03	2.24	0.033					1.46	0.99	2.16	0.057

Model 1: Univariate analysis

Model 2: Age-adjusted analysis

Model 3: Educational attainment, equivalized income, depression, self-rated health, IADL, body mass index, present illness related falls, social participation, walking in min/day, alcohol drinking status and population density were added to model 2. Oral health variables were not simultaneously included.

Model 4: Adjusted variables in model 3 and oral health variables were simultaneously included.

Model 5: All variables are included into the same model.

Table 3 shows the ORs (95% CIs) for female fallers according to oral health variables in the five multilevel logistic regression models. In Models 1–3, all oral health variables were significantly associated with incident falls. In Model 4, difficulty eating tough foods and choking were significantly associated with incident falls; however, no significant association was observed for dry mouth. In Model 5, choking had a significantly high OR (1.64; 95% CI: 1.27–2.11); however, no significant association was found between dry mouth and incident falls. The statistical significance for difficulty eating tough foods was marginal (p<0.10). Furthermore, compared with females having ≥20 teeth, those having 10–19 teeth without dentures, ≤9 teeth with dentures, and ≤9 without dentures had significantly increased risk for incident falls, at 1.63-fold (95% CI: 1.14–2.31), 1.36-fold (95% CI: 1.03–1.80), and 1.46-fold (95% CI: 1.02–2.08), respectively.

**Table 3:** Odds ratios and their 95% confidence intervals of oral health variables in the adjusted multilevel logistic regression models in females.

		Model 1				Model 2				Model 3				Model 4				Model 5			
		OR	95% CI		p	OR	95% CI		p	OR	95% CI		p	OR	95% CI		p	OR	95% CI		p
			Low	High			Low	High			Low	High			Low	High			Low	High	
Difficulty eating tough foods (reference: No)	Yes	1.92	1.55	2.37	<0.001	1.73	1.39	2.14	<0.001	1.41	1.13	1.75	0.002	1.32	1.06	1.65	0.015	1.25	0.99	1.57	0.058
	Data missing	1.66	1.09	2.52	0.018	1.55	1.02	2.36	0.042	1.23	0.70	2.18	0.467	1.13	0.52	2.43	0.762	1.14	0.52	2.46	0.748
Dry mouth (reference: No)	Yes	1.90	1.52	2.39	<0.001	1.75	1.39	2.20	<0.001	1.38	1.08	1.75	0.009	1.21	0.95	1.54	0.130	1.21	0.95	1.54	0.132
	Data missing	1.37	0.93	2.02	0.115	1.26	0.85	1.87	0.246	0.93	0.57	1.51	0.756	0.60	0.30	1.17	0.135	0.61	0.31	1.20	0.149
Choking (reference: No)	Yes	2.23	1.75	2.83	<0.001	2.06	1.61	2.62	<0.001	1.73	1.35	2.21	<0.001	1.63	1.26	2.09	<0.001	1.64	1.27	2.11	<0.001
	Data missing	1.83	1.25	2.69	0.002	1.72	1.17	2.53	0.006	1.55	0.92	2.59	0.097	2.07	1.04	4.12	0.037	2.07	1.04	4.13	0.038
Dental status (reference: ≥20 teeth)	10–19 teeth with dentures	1.36	0.98	1.89	0.069	1.29	0.92	1.79	0.135	1.25	0.90	1.74	0.181					1.19	0.85	1.66	0.310
	10–19 teeth without dentures	1.92	1.36	2.71	<0.001	1.83	1.29	2.60	0.001	1.68	1.18	2.38	0.004					1.63	1.14	2.31	0.007
	≤9 teeth with dentures	2.18	1.68	2.83	<0.001	1.69	1.29	2.22	<0.001	1.46	1.11	1.91	0.006					1.36	1.03	1.80	0.030
	≤9 teeth without dentures	2.30	1.64	3.24	<0.001	1.76	1.25	2.50	0.001	1.55	1.09	2.21	0.014					1.46	1.02	2.08	0.038
	Data missing	1.60	1.08	2.36	0.019	1.22	0.82	1.81	0.325	0.97	0.65	1.45	0.875					0.91	0.61	1.38	0.670

Model 1: Univariate analysis

Model 2: Age-adjusted analysis

Model 3: Educational attainment, equivalized income, depression, self-rated health, IADL, body mass index, present illness related falls, social participation, walking in min/day, alcohol drinking status and population density were added to model 2. Oral health variables were not simultaneously included.

Model 4: Adjusted variables in model 3 and oral health variables were simultaneously included.

Model 5: All variables are included into the same model.

## Discussion

The results of the present study suggest that older people reporting poor oral function, including difficulty eating tough foods, dry mouth, and choking, are more likely to experience falls. These results agree with those from a previous cross-sectional study showing associations between the three questions and falls [13], and further clarify the temporal relationship between poor oral function and incident falls. In the Japanese long-term care insurance system, three questions are used to screen subjects with poor oral function and encourage participation in care prevention services [12]. The results from the present study suggest that the integration of these questions into those regarding fall risk could improve the accuracy of the assessments. Further studies are needed to determine whether improvement of oral function may reduce the risk of falls.

Several possible explanations for the association between poor oral function and incident falls can be envisaged. First, there may be other underlying factors between poor oral health and incident falls, although some adjustments for possible confounders have already been made. For example, some medications are associated with dry mouth [28], and patients treated with such medications are more likely to experience falls [6]. The variable, self-reported present illnesses related to dry mouth, which includes cancer, heart disease, hypertension, diabetes, obesity, hyperlipidemia, respiratory illness, gastrointestinal illness, mental illness, urinary disease and sleep disorder [28], was added to Models 3–5 in consideration of the effects of medication on the association between dry mouth and falls; however, no significant changes were observed in the ORs for dry mouth (data not shown). Further studies using patients’ medication data are necessary to examine the possibility of residual confounders.

Neuromuscular disorders may also represent an underlying factor because they can cause symptoms such as swallowing disorders [29] and an increased risk of falls [6]. To address this issue, two variables of physical activity, ascertained by asking the participants “Do you go upstairs without holding on to the handrail or the wall?” and “Do you get up out of a chair without holding anything?”, with possible answers dichotomized into yes and no [22], were added to Models 3–5. No significant changes were observed in the ORs for difficulty eating tough foods or choking (data not shown); however, further studies using data regarding neuromuscular conditions are needed to address the possibility of residual confounders.

Second, a decline in oral function may be part of a self-perpetuating cycle of frailty [30], which could in turn increase the risk of falls. Several studies have reported associations between chewing ability and physical fitness, such as lower extremity dynamic strength and equilibrium [31], which is a primary cause of falls [32]. One review showed that swallowing or chewing problems and poor oral intake were associated with an increased likelihood of weight loss [33]. We were unable to consider all potential confounders regarding general frailty because the present study was observational. Although further studies are necessary to examine the causal associations between incident falls and dry mouth and choking, the findings suggest that these indicators could be used as predictors.

Another possibility is that poor oral function could increase the degree of risk factors for falls, such as depression. One recent longitudinal study reported that older people who experienced more difficulty chewing tough foods developed depressive symptoms [16], which is a risk factor for incident falls [7,21]. However, depression was adjusted as a covariate in the present study, so other pathways may be involved.

In another previous study, analyses were not conducted separately by sex because of the relatively small sample size [7]. Balance performance in females was worse than that in males among older people [34, 35], suggesting that the effects of dental status on incident falls may be significant in females, but not in males. The ORs for females having 10–19 teeth without dentures, but not for those having 10–19 teeth with dentures, were significantly high. These results agree with those of a previous study [7], even though in that study, 10–19 teeth and ≤9 teeth were combined in the same category.

The ORs for three oral function variables in Model 4 were lower than were those in Model 3, indicating an association between these variables. In addition, the ORs for difficulty eating tough foods and dental status in Model 5 were lower than were those in Model 3, indicating an association. From the results of present study, we could not conclude whether oral function and dental status were the cause of falls, or whether they were mediators between systemic conditions and falls. However, our results do show that poor oral function and dental status significantly increased the risk of falls, even after adjusting for variables in relation to systemic conditions. Therefore, we consider that in addition to the effect of systemic conditions, oral function and dental status, especially occlusion, exert a direct effect on incident falls. One possible explanation for the mechanism between falls and occlusion is the effect of jaw position on body posture [36]. Proprioceptive receptors of the masticatory muscular system and dentoalveolar ligaments provide sensory afferent input [37]; hence, poor dental occlusion may decrease that proprioception, thereby interfering with the stability of head posture and increasing the risk of falls. In fact, one longitudinal study showed that partial or complete loss of dental occlusion was associated with a decline in lower extremity dynamic strength and balance function [9], and a clinical study showed that denture use improves balance and control in older people [38].

The primary strength of this study was its large sample size, population-based sampling, and control for numerous confounding factors. In addition, a wide range of municipalities was surveyed to consider regional differences in incident falls [14].

This study did have several limitations. First, oral health status was based on self- rather than clinical assessments. However, the validity and reliability of self-assessed oral health status has been established and widely used in epidemiological studies [39]. Furthermore, the validity of our questionnaire for dental status has been confirmed [40]. Second, self-reported falls may not be completely factual. However, the associations between falls and demographic factors and other covariates were in the generally expected direction, suggesting that there may be sufficient value in these outcomes.

## Conclusions

This longitudinal study using data from community-dwelling older people showed that poor oral function, including difficulty eating tough foods, dry mouth, and choking, was associated with incident falls. Moreover, having fewer teeth and not using dentures were independent predictors of falls in older females. Further studies are needed to determine whether improvement of oral health can reduce the risk of falls.
